# Synthesis, Characterization, and Antimicrobial Evaluation of Oxadiazole Congeners

**DOI:** 10.3390/molecules16064339

**Published:** 2011-05-25

**Authors:** Bassem Sadek, Khairi Mustafa Salem Fahelelbom

**Affiliations:** Department of Pharmaceutical Sciences, College of Pharmacy, Al-Ain University of Science and Technology, P.O. Box 64141, Al Ain, United Arab Emirates

**Keywords:** 1,3-oxazole, oxadiazole congeners, 1,3-thiazole, tetrazole, antibacterial, antifungal

## Abstract

A series of 1,3-oxazole, 1,3-thiazole, isomeric 1,2,4-oxadiazole, 1,3,4-oxadiazole, and 1,2,3,4-tetrazole heterocycles was synthesized. All the compounds shared as a common feature the presence of a 4-hydroxyphenyl substituent. The structures of the synthesized compounds were confirmed by MS, ^1^H-NMR, and elemental analysis. *In vitro* antimicrobial activity for all the newly synthesized compounds at concentrations of 200-25 μg/mL was evaluated against Gram+ve organisms such as *methicillin-resistant Staphylococcus aureus* (*MRSA*), Gram–ve organisms such as *Escherichia coli* (*E. coli*), and the fungal strain *Aspergillus niger* (*A. niger*) by the cup plate method. Ofloxacin and ketoconazole (10 μg/mL) were used as reference standards for antibacterial and antifungal activity, respectively. Compounds **15**, **16**, and **20** showed notable antibacterial and antifungal activities at higher concentrations (200 μg/mL), whereas **17**-**19** were found to display significant antibacterial or antifungal activity (25-50 μg/mL) against the Gram+ve, Gram–ve bacteria, or fungal cells used in the present study.

## 1. Introduction

Heterocycles containing nitrogen(s) and an oxygen or sulfur atom constitute an important class of compounds in the field of medicinal chemistry due to their interesting and diverse clinical applications [1]. For example, considerable evidence has accumulated during the past two decades demonstrating the various pharmacological effects of 1,3,4-oxadiazoles, which include antibacterial [1], antifungal [2], anthelmintic [3], antitubercular [4], anticancer [5], anti-HIV [6], antioxidant [7], analgesic [8], anti-inflammatory [9] and anticonvulsant [10] activities. In spite of the large number of antibiotics and chemotherapeutics currently available for medical usage, the spectre of increasing bacterial resistance has made it necessary to continue the search for new antimicrobial substances. To this end a large number of oxadiazole derivatives have been prepared, many of which have shown a wide spectrum of antimicrobial activity. Some oxadiazoles with different substituents, especially with a 4-hydroxy-phenyl moiety at different locations on the five-membered heterocyclic ring, produced fungicidal and bactericidal agents of various potencies [11,12]. These observations promoted us to develop, synthesize, and evaluate a novel series of diverse heterocycles of more simplified chemical structures that include 1,3-oxazole, 1,3-thiazole, isosteric oxadiazoles, and tetrazole heterocyclic ring systems, all bearing a 4-hydroxyphenyl moiety.

## 2. Results and Discussion

### 2.1. Synthesis

**Scheme 1:**
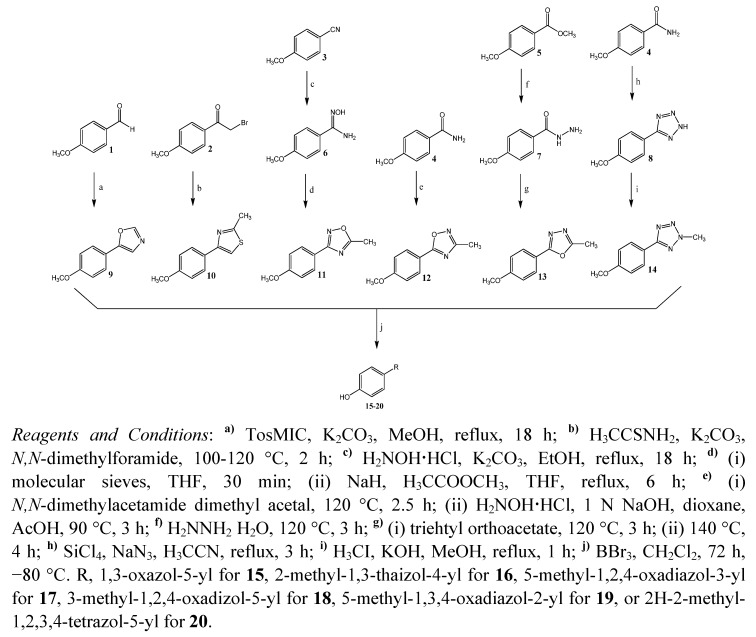
Synthesis of heterocyclic derivatives **15**-**20**.

All novel heterocyclic compounds were synthesized by ring-closing reactions, with each derivative being prepared *via* a different synthetic pathway (Scheme 1). Key intermediates were the 4-substituted phenylmethyl ethers **9**-**14**. Carbonyl compounds can be efficiently converted into 5-substituted oxazoles in a one-pot reaction with tosylmethyl isocyanide (TosMIC) [13]. Here, the oxazole compound **9** was obtained by reaction of equimolar quantities of TosMIC and the benzaldehyde precursor **1** with potassium carbonate in refluxing methanol (Scheme 1). 

The most common and most versatile procedure for the formation of 1,3-thiazoles is the cyclocondensation of α-haloketones with appropriate thioamide derivatives [14]. In this study, the reaction of commercially available α-bromoketone **2** with thioacetamide under basic conditions furnished the 4-substituted heterocyclic derivative **10**. The amide oxime **6** provided the starting point for the synthesis of the 3,5-disubstituted 1,2,4-oxadiazole **11**, which was generated by reaction of hydroxylamine, liberated from its hydrochloride using potassium carbonate, with *p*-anisonitrile **3** in refluxing ethanol.

Dehydration, cyclization, and aromatization by subsequent treatment with acetic acid methyl ester in the presence of sodium hydride in tetrahydrofuran afforded the corresponding 5-methyl-1,2,4-oxadiazole compound **11** [15]. The 3-methyl-1,2,4-oxadiazole derivative **12** was prepared adapting the procedure described by Lin *et al*. [16]. Thus, reaction of the carboxamide **4** with *N,N*-dimethylacetamide dimethyl acetal and cyclization of the intermediate acylamidine with hydroxylamine provided **12** in good yield.

According to the procedure described by Ainsworth, synthesis of the 5-methyl-1,3,4-oxadiazole derivative **13** was achieved by treatment of the ester **5** with hydrazine to afford the hydrazide intermediate **7** followed by cyclization with triethyl orthoacetate [17]. In a direct one-pot reaction, primary amide **4** was converted with triazidochlorosilane into the 5-substituted tetrazole intermediate **8** [18]. Adapting the protocol of Begtrup and Larsen [19], compound **8** was methylated with methyl iodide in methanolic potassium hydroxide. The resulting mixture of isomeric 1-methyl- and 2-methyl-tetrazole derivatives was separated by column chromatography and showed a product ratio of 4:1 in favor of **14**. The ^1^H- and ^13^C-NMR data for **14** were in full agreement with literature data [19,20,22]. Finally, proceeding from the different heterocyclic derivatives **9**-**14**, methyl ether cleavage with BBr_3_ in dichloromethane provided the corresponding phenols **15**-**20** in high yields [21].

### 2.2. Biological Activity

The 1,3-oxazole derivative **15** and its bioisoster and the sulfur-containing 1,3-thiaziole derivative **16** exhibited low antimicrobial activity (MIC 200 μg/mL) compared to the reference substances ofloxacin and ketoconazole. Major improvement in antimicrobial activity was obtained with the next three compounds, the methyloxadiazole derivatives **17**-**19**. Within this homogenous series, the oxadiazoles differ from each other solely in the position of the three heteroatoms of the five-membered aromatic ring with regard to the location of the methyl or 4-hydroxyphenyl moiety. The 1,2,4-oxadiazole derivative **17** having a 4-hydroxyphenyl at the 3-position of 5-methyl-1,2,4-oxadiazole exhibited a MIC of 25 μg/mL against *S. aureus* and *A. niger*, whereas **18** having the 4-hydroxyphenyl at the 5-position of 3-methyl-1,2,4-oxadiazole exhibited significant antimicrobial activity (MIC 25 μg/mL) against *E. coli*, and *A. niger*. The bioisosteric 5-methyl-1,3,4-oxadiazole **19** exhibited promising antibacterial activity (MIC 25 μg/mL) against all tested culture strains, indicating that changes in the relative positions of the heteroatoms inside the heterocycle rings influences the antimicrobial activity. Since a carboxylic acid group can bioisosterically be replaced by a tetrazole, the next compound **20** could be interpreted as a potential bioisoster of the corresponding benzoic acid methyl ester derivative, yet the 2-methyl-2*H*-tetrazole derivative **20** failed to exhibit high antimicrobial activity (MIC 200 μg/mL) against *S. aureus*, *E. coli* or *A. niger* (Table 1).

**Table 1:** Antimicrobial activity of the title compounds. 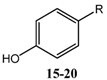

	MIC µg/mL			
Compound	R	*S. aureus*	*E. coli*	*A. niger*
**15**	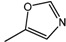	200	200	200
**16**	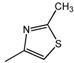	150	200	150
**17**	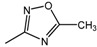	25	50	25
**18**	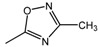	50	25	25
**19**	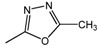	25	25	25
**20**	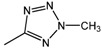	200	200	200
**Ofloxacin**		10	12.5	--
**Ketoconazole**		--	--	12.5

## 3. Experimental

### 3.1. General Procedures

Melting points are uncorrected and were determined in open capillaries in a Büchi 512 Dr. Tottoli apparatus. ^1^H-NMR spectra were recorded on a Bruker WC 300 spectrometer with tetramethylsilane (TMS) as internal standard. Chemical shifts are reported in ppm downfield from internal tetramethylsilane as reference. ^1^H-NMR signals are reported in order: multiplicity (s, single; d, doublet; t, triplet; m, multiplet; *, exchangeable by D_2_O), number of protons, and approximate coupling constants expressed in Hertz. For compound **20**, a ^13^C-NMR spectrum (100 MHz) was recorded on a Bruker DPX 400 Avance instrument, and chemical shifts that distinguish whether a methyl group is placed at the 1- or 2-position of the tetrazole moiety are reported in ppm downfield from internal TMS used as reference. Elemental analyses were performed on Perkin-Elmer 240B and 240C instruments. Analyses (C, H, N) indicated by the symbols of elements were within ±0.4% of the theoretical values. Chromatographic separations were done using a Chromatotron Model 7924 (Harrison Research) with 4 mm layers of silica gel 60 PF containing gypsum (Merck). EI-mass spectra were recorded using Finnigan MAT CH7A (70 eV), Finnigan MAT 711 (80 eV), or Kratos MS 25 RF (70 eV) instruments. ^+^FAB-MS spectra were recorded on Finnigan MAT CH5DF instrument (xenon, DMSO)/glycerol). The MS spectra (data not shown) corresponded in all cases with the structures of the compounds depicted in the reaction schemes. The following abbreviations are used: Benz, benzoxathiazolyl; EtOH, ethanol; Et_2_O, diethyl ether; MeOH, methanol; Me_2_SO, dimethyl sulfoxide; Ox, oxadiazolyl; Ph, phenyl; Tet, tetrazolyl; Th, thiazolyl; THF, tetrahydrofuran. 

*Methyl 4-(1,3-oxazol-5-yl)phenyl ether* (**9**): *p*-Toluenesulfonylmethyl isocyanide (0.27 g, 1,4 mmol) and **1** (0.19 g, 1.4 mmol) were dissolved in dry MeOH (30 mL). Next K_2_CO_3_ (0.19 g, 1.4 mmol) was added slowly over 30 min. After refluxing for 18 h the solvent was concentrated under vacuum and the residual aqueous layer was extracted with CH_2_Cl_2_. The organic extracts were combined, dried, and concentrated under reduced pressure. The oily product was purified by column chromatography (eluent: CH_2_Cl_2_) and crystallized at room temperature: Yield 61%; mp 61-63 °C; ^1^H-NMR δ ppm = 3.83 (s, 3H, H_3_CO), 7.02 (d, *J* = 8.6 Hz, 2H, Ph-3,5H), 7.52 (s, 1H, Ox-4H), 7.64 (d, *J* = 8.6 Hz, 2H, Ph-2,6H), 8.36 (s, 1H, Ox-2H); EI-MS m/z (%) 176 (M^+^, 21).

*Methyl 4-(2-methyl-1,3-thiazol-4-yl)phenyl ether* (**10**): 4-Methoxyphenacyl bromide (**2**, 1.49 g, 6.5 mmol), thioacetamide (0.48 g, 6.5 mmol), and K_2_CO_3_ (1.20 g, 8.7 mmol) were dissolved in dry *N,N*-dimethylformamide (30 mL). The mixture was stirred for 2 h at 100-120 °C, cooled, and the solvent was removed under reduced pressure. The solid residue was recrystallized from EtOH. Yield 79%; mp 59-60 °C; ^1^H-NMR δ ppm = 2.70 (s, 3H, Th-CH_3_), 3.79 (s, 3H, H_3_CO), 6.99 (d, *J* = 8.7 Hz, 2H, Ph-3,5H), 7.74 (s, 1H, Th-5H), 7.87 (d, *J* = 8.7 Hz, 2H, Ph-2,6H); EI-MS m/z (%) 205 (M^+^, 100).

*Methyl 4-(5-methyl-1,2,4-oxadiazol-3-yl)phenyl ether* (**11**): *p*-Anisonitrile (**3**, 6.66 g, 50 mmol), hydroxylamine hydrochloride (6.95 g, 100 mmol), and K_2_CO_3_ (10.34 g, 75 mmol) in dry EtOH (80 mL) were heated under reflux for 18 h. The mixture was cooled, filtered, and concentrated under reduced pressure. The crystalline residue was suspended in MeOH, and the final product **6** was isolated by filtration and dried. Powdered molecular sieves (4 Ǻ, 4 g) were added to a solution of **6** (1.66 g, 10 mmol) in dry THF (60 mL). After stirring for 30 min NaH (suspended in mineral oil, ω = 60%, 0.22 g, 5.5 mmol) was added, and the mixture was heated for 45 min at 60 °C. After cooling acetic acid methyl ester (1.48 g, 20 mmol) dissolved in dry THF was dropwise. Subsequently, the mixture was refluxed for 6 h under anhydrous conditions. Cooling, filtration, and removal of the solvent under reduced pressure resulted in a solid residue **11** which was recrystallized from EtOH. Yield 83%; mp 61-62 °C; ^1^H-NMR δ ppm = 2.64 (s, 3H, Ox-CH_3_), 3.83 (s, 3H, H_3_CO), 7.11 (d, *J* = 8.8 Hz, 2H, Ph-3,5H), 7.92-7.95 (m, 2H, Ph-2,6H); FAB^+^-MS m/z (%) 191 (M^+^ + H^+^, 100).

*Methyl 4-(3-methyl-1,2,4-oxadiazol-5-yl)phenyl ether* (**12**): A solution of 4-methoxybenzamide (**4**, 1.01 g, 6.7 mmol) in *N,N*-dimethylacetamide dimethyl acetal (15 mL) was stirred for 2.5 h at 120 °C under N_2_. The solvent was removed under reduced pressure and hydroxylamine hydrochloride (0.65 g, 9.4 mmol) dissolved in an aqueous NaOH solution (9.14 mL, c = 1 mol/L) was added. After addition of dioxane (10 mL) and acetic acid (12.5 mL) the mixture was stirred for 1.5 h at room temperature. Subsequently, the mixture was heated for 3 h at 90 °C, cooled, and a saturated solution of K_2_CO_3 _in H_2_O was added. Then, the mixture was concentrated under vacuum and the residual aqueous layer was extracted with CH_2_Cl_2_. The organic extracts were combined, dried, and concentrated under reduced pressure. The oily product **12** was purified by column chromatography (eluent: CH_2_Cl_2_) and crystallized at 4 °C. Yield 66%; mp 59-61 °C; ^1^H-NMR δ ppm = 2.39 (s, 3H, Ox-CH_3_), 3.87 (s, 3H, H_3_CO), 7.15-7.18 (m, 2H, Ph-3,5H), 8.03 (d, *J* = 8.8 Hz, 2H, Ph-2,6H); EI-MS m/z (%) 190 (M^+^, 92).

*Methyl 4-(5-methyl-1,3,4-oxadiazol-2-yl)phenyl ether* (**13**): A mixture of 4-methoxybenzoic acid methyl ester (**5**, 4.99 g, 30 mmol) and hydrazine monohydrate (3.00 g, 60 mmol) was heated at 120 °C for 3 h. Cooling and dilution with H_2_O (10 mL) afforded a precipitate of compound **7** which was filtered, washed with H_2_O, and dried *in vacuo*. Triethyl orthoacetate (10 mL) and **7** (1.66 g, 10 mmol) were heated at 120 °C for 3 h. Excess orthoacetate was evaporated, and the residue heated for further 2 h at 140 °C. The reaction was diluted with H_2_O (10 mL), saturated with K_2_CO_3_, and extracted with Et_2_O. Evaporation of the dried extracts afforded **13** as an oil. Yield 66%; mp 88°C; ^1^H-NMR δ ppm = 2.56 (s, 3H, Ox-CH_3_), 3.85 (s, 3H, H_3_CO), 7.91 (d, *J* = 8.8 Hz, 2H, Ph-3,5H), 7.91 (d, *J* = 8.7 Hz, 2H, Ph-2,6H); EI-MS m/z (%) 190 (M^+^, 93).

*Methyl 4-(2-methyl-2H-tetrazol-5-yl)phenyl ether* (**14**): A suspension of silicon(IV) chloride (9.35 g, 55 mmol) and sodium azide (7.15 g, 110 mmol) in dry acetonitrile (20 mL) was stirred for 1 h at ambient temperature. 4-Methoxybenzamide (**4**, 4.00 g, 27 mmol) was added dropwise. After stirring under for 3 h, the reaction mixture was poured into ice-cooled solution of K_2_CO_3 _in H_2_O, filtered, and acidified with aqueous HCl. The precipitated product **8** was filtered, washed with H_2_O, and dried *in vacuo*. A solution of **8 **(1.86 g, 10.6 mmol) and KOH (2.81 g, 50 mmol) in dry MeOH (30 mL) was mixed at -25 °C with iodomethane (4.56 g, 32.1 mmol) in dry MeOH (5 mL). The temperature was raised to 20 °C during 1 h and the mixture refluxed for an additional hour. The solvent was removed and the crude product **14** purified by column chromatography (eluent: CH_2_Cl_2_). Yield 60%; mp 56-57 °C; ^1^H-NMR δ ppm = 3.83 (s, 3H, H_3_CO), 4.40 (s, 3H, NCH_3_), 7.10-7.13 (m, 2H, Ph-3,5H), 7.97-8.00 (m, 2H, Ph-2,6H); ^13^C-NMR (CDCl_3_) δ 39.7 (NCH_3_), 55.8 (H_3_CO), 114.7 (Ph-2,6C), 120.4 (Ph-4C), 128.7 (Ph-3,5C), 161.7 (Ph-1C), 165.6 (Tet-5C); EI-MS m/z (%) 190 (M^+^, 21).

### 3.2. General Procedure for Ether Cleavage

A solution of the corresponding ether (4 mmol) in dry CH_2_Cl_2_ (20 mL) was cooled to −80 °C under exceeding −60 °C. The reaction mixture was then allowed to warm to room temperature and stirred for additional 72 h. Subsequently, the mixture was cooled to −80 °C and MeOH (25 mL) was added dropwise. The organic layer was removed from the mixture under reduced pressure. After addition of a saturated K_2_CO_3_ solution in H_2_O to the aqueous layer, the crude product precipitated. It was isolated by filtration and recrystallized from EtOH.

*4-(1,3-Oxazol-5-yl)phenol* (**15**): From **9.** Yield 41%; mp 43-45 °C; ^1^H-NMR δ ppm = 7.02 (d, *J* = 8.6 Hz, 2H, Ph-3,5H), 7.52 (s, 1H, Ox-4H), 7.64 (d, *J* = 8.6 Hz, 2H, Ph-2,6H), 8.36 (s, 1H, Ox-2H), 9.55 (s*, 1H, OH); EI-MS m/z (%) 161 (M^+^, 21). Anal. (C_9_H_7_NO_2_): C, H, N calcd. 67.07, 4.38, 8.69; found. 67.05, 4.26, 8.56.

*4-(2-Methyl-1,3-thiazol-4-yl)phenol* (**16**): From **10**. Yield 70%; mp 151 °C; ^1^H-NMR δ ppm = 2.69 (s, 3H, Th-CH_3_), 6.81 (d, *J* = 8.6 Hz, 2H, Ph-3,5H), 7.64 (s, 1H, Th-5H), 7.55 (d, *J* = 8.6 Hz, 2H, Ph-2,6H), 10.45 (s*, 1H, OH); EI-MS m/z (%) 191 (M^+^, 100). Anal. (C_10_H_9_NOS): C, H, N calcd. 62.80, 4.74, 7.32; found. 62.31, 4.52, 7.02.

*4-(5-Methyl-1,2,4-oxadiazol-3-yl)phenol* (**17**): From **11**. Yield 84%; mp 189-191 °C; ^1^H-NMR δ ppm = 2.62 (s, 3H, CH_3_), 6.89-6.92 (m, 2H, Ph-3,5H), 7.92 (d, *J* = 8.7, 2H, Ph-2,6H), 10.55 (s*, 1H, OH); EI-MS m/z (%) 176 (M^+^, 100). Anal. (C_9_H_8_N_2_O_2_): C, H, N calcd. 61.36, 4.58, 15.90; found. 61.34, 4.49, 15.48.

*4-(3-Methyl-1,2,4-oxadiazol-5-yl)phenol* (**18**): From **12**. Yield 83%; mp 186 °C; ^1^H-NMR δ ppm = 2.37 (s, 3H, CH_3_), 6.94-6.98 (m, 2H, Ph-3,5H), 7.92 (d, *J* = 8.7 Hz, 2H, Ph-2,6H), 10.49 (s*, 1H, OH); EI-MS m/z (%) 176 (M^+^, 69). Anal. (C_9_H_8_N_2_O_2_): C, H, N calcd. 61.36, 4.58, 15.50; found. 61.33, 4.47, 15.43.

*4-(5-Methyl-1,3,4-oxadiazol-2-yl)phenol* (**19**): From **13**. yYield 55%; mp 236 °C; ^1^H-NMR δ ppm = 2.56 (s, 3H, CH_3_), 6.98 (d, *J* = 8.6 Hz, 2H, Ph-3,5H), 7.85 (d, *J* = 8.6 Hz, 2H, Ph-2,6H), 10.26 (s*, 1H, OH); EI-MS m/z (%) 176 (M^+^, 100). Anal. (C_9_H_8_N_2_O_2_): C, H, N calcd. 61.36, 4.58, 15.50; found. 61.33, 4.44, 15.43.

*4-(2-Methyl-2H-tetrazol-5-yl)phenyl ether* (**20**): From **14**. Yield 95%; mp 210-212 °C; ^1^H NMR δ ppm = 4.38 (s, 3H, NCH_3_), 6.92 (d, *J* = 8.6 Hz, 2H, Ph-3,5H), 7.87 (d, *J* = 8.5 Hz 2H, Ph-2,6H), 9.96 (s*, 1H, OH); EI-MS m/z (%) 176 (M^+^, 22). Anal. (C_8_H_8_N_4_O): C, H, N calcd. 54.54, 4.58, 31.80; found. 54.49, 4.41, 31.78.

### 3.3. Antimicrobial Activity

The quantitative *in vitro* antimicrobial study was carried on Muller-Hinton agar (Hi-media) plates (37 °C, 24 h) by the agar diffusion cup plate method [23]. The compounds (200-25 μg/mL) were screened for antimicrobial activity against the bacterial strains *Staphylococcus aureus* ATCC 25923 (*S. aureus*) (Gram+ve) and *Escherchia coli* ATCC 35218 (*E. coli*) (Gram-ve). Antifungal activity was tested on Sabouraud dextrose agar (Hi-media) plates (26 °C, 48-72 h) by the cup plate method against *Aspergillus niger* A733 (*A. niger*) also at a concentration level of 200-25 μg/mL. Ofloxacin and ketoconazole were used as standards for comparison of antibacterial and antifungal activity under the similar conditions. DMF was used as a solvent control for both antibacterial and antifungal activities, and the results are presented in minimal inhibition concentration (MIC) values (μg/mL) in Table 1. 

## 4. Conclusions

The new compounds **15**-**20** presented here clearly differ in their corresponding antimicrobial activity depending on the type of the heterocycle. In the course of this study, particularly the derivatives possessing methyloxadiazole moieties **17**-**19** were identified as possessing moderate antibacterial activity against *methicillin-resistant* *S. aureus* (Gram positive) and *E. coli* (Gram negative) bacteria and antifungal activity against *A. niger*. These results, combined with the potential benefits or at least differences in pharmacokinetics make the titled oxadiazole congeners not only interesting simplified leads for the further chemical optimization of this class but also potentially interesting for future scope to study their mechanism of action and would be worthy of additional structure-activity relationship investigation.
